# Patients’ perspectives on the benefits of feedback on patient-reported outcome measures in a web-based personalized decision report for hip and knee osteoarthritis

**DOI:** 10.1186/s12891-022-05764-1

**Published:** 2022-08-23

**Authors:** Brocha Z. Stern, Sarah Pila, Layla I. Joseph, Nan E. Rothrock, Patricia D. Franklin

**Affiliations:** 1 Center for Education in Health Sciences, Northwestern University Feinberg School of Medicine, Chicago, IL USA; 2 Department of Population Health Science and Policy, Icahn School of Medicine at Mount Sinai, 1 Gustave L. Levy Place, New York, NY 10029 USA; 3 Department of Medical Social Sciences, Northwestern University Feinberg School of Medicine, Chicago, IL USA

**Keywords:** Arthroplasty, Digital health, Osteoarthritis, Orthopedic surgery, Patient engagement, Patient-reported outcomes, Qualitative research

## Abstract

**Background:**

Applications of patient-reported outcome measures (PROMs) for individual patient management are expanding with the support of digital tools. Providing PROM-based information to patients can potentially improve care experiences and outcomes through informing and activating patients. This study explored patients’ perspectives on the benefits of receiving feedback on PROMs in the context of a web-based personalized decision report to guide care for their hip or knee osteoarthritis.

**Methods:**

This qualitative descriptive interview study was nested in a pragmatic clinical trial of a personalized report, which includes descriptive PROM scores and predicted postoperative PROM scores. Patients completed a semi-structured interview within 6 weeks of an office visit with an orthopaedic surgeon. Only patients who reported receiving the report and reviewing it with the surgeon and/or a health educator were included. Data were iteratively analyzed using a combination of deductive and inductive coding strategies.

**Results:**

Twenty-five patients aged 49–82 years (60% female, 72% surgical treatment decision) participated and described three primary benefits of the PROM feedback within the report: 1. Gaining Information About My Health Status, including data teaching new information, confirming what was known, or providing a frame of reference; 2. Fostering Communication Between Patient and Surgeon, encompassing use of the data to set expectations, ask and answer questions, and facilitate shared understanding; and 3. Increasing My Confidence and Trust, relating to the treatment outcomes, treatment decision, and surgeon.

**Conclusions:**

Patients identified actual and hypothetical benefits of receiving feedback on PROM scores in the context of a web-based decision report, including advantages for those who had already made a treatment decision before seeing the surgeon. Findings provide insight into patients’ perspectives on how digital PROM data can promote patient-centered care. Results should be considered in the context of the homogeneous sample and complex trial. While participants perceived value in this personalized report, questions remain regarding best practices in patient-facing data presentation and engagement.

**Trial registration:**

ClinicalTrials.gov, NCT03102580. Registered on 5 April 2017.

**Supplementary Information:**

The online version contains supplementary material available at 10.1186/s12891-022-05764-1.

## Background

Patient-reported outcome measures (PROMs) capture multidimensional consequences of hip and knee osteoarthritis that matter to patients, such as symptoms and the functional, emotional, and social impact of living with a chronic condition [1, 2]. Routine collection of PROMs has become the standard of care in total hip/knee arthroplasty (THA/TKA), with digital tools supporting large-scale data collection. Applications for quality reporting and learning health systems research are proposed to accelerate value-based care [3, 4], and applications for shared decision making and postoperative monitoring are advocated for individual patient management [5]. While a review identified limited research on clinical practice integration of PROMs for hip and knee osteoarthritis [6], subsequent publications highlight emerging innovations related to digital PROM-based decision aids or feedback reports in THA/TKA [7–13].

Clinicians’ and patients’ perceived benefits of using PROMs for individual patient management include patient involvement and personalized, holistic care [14]. Patient-facing PROM digital tools may support patient-clinician communication [15] and improve management of THA/TKA expectations [16], potentially increasing patient satisfaction with care experiences and health outcomes [17]. However, one study of a PROM-based feedback report in patients with hip or knee osteoarthritis found no meaningful between-group differences in patient activation, satisfaction, or perceptions of the physician–patient relationship [18]. A Cochrane review on PROM feedback across multiple clinical conditions also identified limited to no improvements in patient perceptions of self-efficacy, unmet needs, or satisfaction [15].

At the health system level, the chronic care model posits that implementing PROM-based feedback applications may facilitate interactions between informed, active patients and prepared, proactive care teams [19]. However, mixed findings of benefits of PROM-based feedback warrant further exploration of patients’ perspectives in the context of THA/TKA. We, therefore, embedded a qualitative interview component in an ongoing trial of a web-based personalized decision report to guide care for hip or knee osteoarthritis [8]. Decision aids broadly aim to help patients make informed choices and can be reviewed alone and/or with a clinician [20]. Web-based interfaces can support individualized report content [21]. The report in this trial provides feedback on PROM data to assist with decision making and is designed to be both patient- and surgeon-facing. This paper specifically explores patients’ perspectives on benefits of receiving this digital PROM feedback in the context of a consultation with an orthopaedic surgeon.

## Methods

### Design

**Fig. 1:**
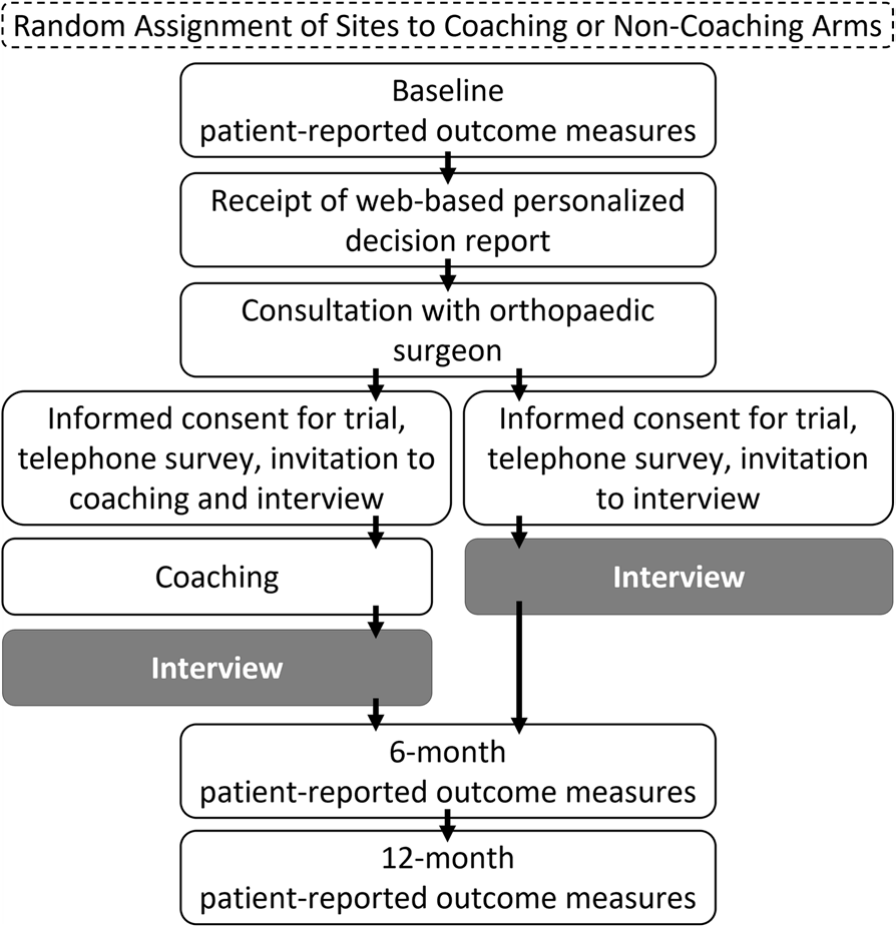
Flow of trial procedures contextualizing timing of interview. Report receipt could be simultaneous with consultation. All interview participants confirmed report receipt on the telephone survey, but a few participants recalled during the interview that they received the report after the office visit. In the non-coaching arm, interviews took place within 4 weeks of the visit. In the coaching arm, interviews took place within 2 weeks of the coaching session, which took place within 4 weeks of the visit

The qualitative interview project was nested in a pragmatic cluster-randomized clinical trial (ClinicalTrials.gov identifier: NCT03102580). Figure 1 presents a flow diagram of trial procedures. Procedures were approved by the University of Massachusetts Medical School Institutional Review Board (H00012297). Patients completed PROMs electronically or with telephone support at a single point before their visit with a participating orthopaedic surgeon related to hip or knee osteoarthritis. Patients may have seen other surgeons previously for diagnosis or treatment recommendations. All patients were considered to have made a treatment decision (surgery versus alternative) during the consultation, with the recognition that the decision may change (e.g., reconsider surgery after weight loss). Some patients consulted a physician assistant per routine workflows, but we did not distinguish between clinician types. PROMs included the Hip disability/Knee injury and Osteoarthritis Outcome Score (HOOS/KOOS-12) [22, 23] and the Veterans RAND 12-Item Health Survey [24]. These PROMs are routinely collected for THA/TKA quality reporting in the United States, and national data are available for analytics [25]. Patients self-reported pain intensity in other hips/knees and low back, sociodemographic factors, and medical comorbidities. As recommended by the United States Centers for Medicare and Medicaid Services, a question on confidence completing health forms screened for health literacy [26].

The personalized PROM-based report (see Additional file 1: Appendix) integrates the patient’s individual scores with real-time data from a national patient-centered registry [25]. Its development has been previously described [8, 27]. A paper copy facilitated point of care use and accommodated diverse patient preferences. However, the report was powered by a web-based interface that supported predictive analytics. Although not required for the trial, sites could file the report digitally within the local electronic health record. Page 1 presents the patient’s current PROM scores (hip and knee pain, hip/knee-related physical function, general physical health) compared to scores in the national sample. It also presents risk factors (e.g., body mass index, emotional health) affecting the condition and postoperative outcomes. Page 2 provides estimates of likely 12-month postoperative self-reported outcomes (hip/knee pain, hip/knee-related physical function, general physical health) based on a multivariable predictive model of similar patients. Page 1 and 2 use numbers and colors (green, orange, red) to present PROM and clinical risk data. Page 3 provides a generic decision grid for nonoperative options and is not this paper’s focus as it is not PROM-based.

Because of dynamic office workflows related to COVID-19, including paperless environments for infection control, some patients received the report via mail/email at home before the surgeon’s visit and others in the office. A few reported receiving it after the office visit likely due to mail delays although some expressed uncertainty with timing of receipt. After the visit, patients were consented to the trial by a research coordinator unaffiliated with the surgeons’ offices. They completed a telephone survey that included their decision (surgery versus alternative), the Decisional Conflict Scale [28], and recall of report receipt and use with the surgeon. Patients at randomly assigned sites were also invited to a coaching session: a post-visit 1-h virtual group session where a health educator reviewed a sample report. Coaching was added to the trial based on feedback from a patient advisory board to support patients in using the data when continuing to discuss treatment options with their medical team.

### Sampling and recruitment

Trial participants were new patients ≥ 40 years of age evaluated by a participating surgeon for hip or knee osteoarthritis. Patients were excluded if they were pregnant, visited the surgeon for a recent hip or knee injury, or had inflammatory arthritis or contraindications to THA/TKA. The qualitative interview sample was limited to English-speaking patients who confirmed report receipt on the telephone survey. To encourage depth of perspectives on the PROM feedback, we required report use with the surgeon per the telephone survey or participation in coaching. However, while 19 participants reported on the telephone survey that they used the report with their surgeon, only 12 confirmed surgeon review during the interview. This discrepancy highlights potential ambiguity with the question or challenges with recall of the content of clinical visits.

Eligible patients were invited to participate in the interview component when they consented to the trial between April-July 2021. We initially estimated a sample size of 25–40 based on information power [29]. The upper estimate was intended to enable comparisons between report exposure categories (use with surgeon, coaching, or surgeon and coaching). Challenges discriminating between report exposure categories based on participants’ telephone survey and interview responses did not support meaningful between-group comparisons, and we deemed information to be adequate at 25.

### Data collection

Each participant completed one semi-structured telephone or video interview (mean 37 min, range 23–69 min) within 6 weeks of the office visit (mean 18 days, range 3–34 days). Interviews were scheduled based on participants’ availability, as close as possible to the office visit in the non-coaching arm and the coaching session in the coaching arm. Participants were emailed another copy of their report before the interview, and the report was screen shared when feasible. The interviewers (BZS, SP) were qualitatively trained researchers who were uninvolved with the report’s design and unaffiliated with the surgeons’ offices. The interview guide included open-ended questions and probes (Table 1). Audio recordings of the interviews were professionally transcribed, and the interviewers de-identified and checked the transcripts for accuracy. The interviewers wrote reflective memos after each interview and intermittently debriefed to promote consistency in interview approaches.

**Table 1:** Overview of interview guide

Domain	Sample questions
History of the condition and point in the decision-making process	• Thinking back to your appointment with your surgeon, what were your primary concerns with your [right/left hip/knee] that led you to see the surgeon? • What treatments, if any, had you received for your [right/left hip/knee] before seeing the surgeon?
Use of the report	• Now, talk me through me your experience with the report in your surgeon’s office • How valuable was the report in making a decision about your [hip/knee] arthritis in the surgeon’s office?
Individual components of the report	• Now, let’s go to page 2, the section labeled “your expected outcomes based on patients like you.” What does this section tell you? • Thinking of the full report, what was the most valuable part for you, and why? What was the least valuable part of the report, and why?
Coaching session (if relevant)	• How did your understanding of the report we just reviewed change—if at all—after participating in the coaching session?
General thoughts	• What additional information would have been valuable in making decisions about your [hip/knee] arthritis care? • Anything else you would like us to know?

### Data analysis

We used a qualitative descriptive approach [30] to identify patterns in the data. This approach was chosen as a flexible method of synthesizing patient perspectives of a health services intervention and its functions, facilitators, and barriers [31]. While qualitative research is inherently interpretive, our primary aim was to describe versus engage in higher-level interpretation [31, 32]. Data analysis occurred simultaneously and iteratively with data collection, and we used Dedoose [33] for data management.

After independent close reading and preliminary coding of eight transcripts, BZS and SP collaboratively developed a codebook including deductive and inductive codes [34]. Deductive codes were based on the interview guide (e.g., “point in decision-making process before saw surgeon,” “suggestion for improvement”) and sections of the report (e.g., “Page 1,” “Page 2”). Inductive codes were based on BZS and SP’s discussions of salient details in the data (e.g., “learning style,” “providing an emotional response”). No theoretical framework guided coding, but we used the concept of an “informed, activated patient” from the chronic care model [19] as an analytic lens. BZS and SP piloted the codebook with a subset of transcripts and met to reconcile discrepant coding and address ambiguous or missing codes. After refining the codebook, BZS coded 100% of transcripts, and SP coded 50% of transcripts purposefully selected for heterogeneity. BZS and SP intermittently met throughout the coding process to resolve discrepant coding and further refine the codebook. Transcripts were iteratively recoded as the codebook evolved. After all transcripts were coded, BZS, SP, and LIJ compared and categorized codes and related excerpts within and between transcripts [34]. Per techniques from framework analysis [35], a matrix of codes by cases guided synthesis into a coherent structure, including themes and subthemes. Strategies for trustworthiness included reflective memos and peer debriefing of emerging results in a multi-disciplinary research group.

## Results

### Participants

Forty of 485 participants who consented to the parent trial in April-July 2021 were invited to the interview since they met all interview eligibility criteria (e.g., report receipt and review with surgeon or health educator) and were not past the allowed interview time frame. Thirteen of the invited patients declined to participate because of lack of interest (*n* = 10) or time (*n* = 3), and two became ineligible post-invitation by missing coaching. Two eligible participants were erroneously not invited.

Twenty-five patients aged 49–82 years (*M* = 67.6 ± 9.2) completed the interview. All were White, Non-Hispanic or Latino/a, and none had limited/marginal health literacy per the screening question. They received care from 13 surgeons at five sites in the United States. Table 2 summarizes participants’ demographic characteristics.

**Table 2:** Participants’ demographics

	*n* (%)
Gender	
Female	15 (60)
Male	10 (40)
Education ^a^	
High school graduate/GED	3 (13)
Trade/technical school or some college	3 (13)
Bachelor’s degree	7 (29)
Graduate work	10 (41)
Other	1 (4)
Primary insurance ^a^	
Medicare	15 (63)
Private	8 (33)
Medicaid	1 (4)
Report exposure ^b^	
Used/discussed/reviewed with surgeon + Coaching	10 (40)
Used/discussed/reviewed with surgeon	9 (36)
Coaching	6 (24)
Joint ^c^	
Hip	13 (54)
Knee	11 (46)
Treatment decision	
Surgery	18 (72)
Alternative treatment	7 (28)

^a^ Data missing on 1 participant

^b^ “Used/Discussed/Reviewed report with surgeon” includes physician assistant and is based on participant responses on post-visit telephone survey

^c^ 1 participant’s data is not reported since completed PROMs for hip but made treatment decision about knee

### Benefits of receiving feedback on PROMs

Patients’ benefits of receiving feedback on PROMs in the context of the personalized decision report were categorized into three themes: 1) Providing Information About My Health Status, 2) Fostering Communication Between Patient and Surgeon, and 3) Building My Confidence and Trust. Table 3 summarizes the themes, subthemes, and illustrative quotes. Parentheses after quotes reflect each participant’s interview ID, age, gender, involved joint, trial arm, and treatment decision.

**Table 3:** Themes, subthemes, and illustrative quotes

Theme	Subtheme	Illustrative Quote
Providing Information About My Health Status	Teaching something new	*“Since I got the shot, my left hip doesn't bother me. So, I mean that I didn't associate it with problems, but this report helped me see that. I just thought the hip is the hip and the knee is the knee. But I imagine if you're having problem in one area, it could affect the other.”* (P10, 70 y.o. Female, Knee, Coaching, Alternative)
	Confirming what know	*“It basically just reiterates what I already knew from, based on how I answered the questions. It was good seeing that my pain is still in the moderate section, so there were other options besides surgery for me at this point.”* (P18, 56 y.o. Female, Knee, Non-Coaching, Alternative)
	Providing frame of reference	*“Because like I said, it's been hurting for so long that it's kind of just like there anymore. But then when you look at the survey and take the answers and do the answers, and then you're like, ‘Wow, that's – it's only a 19, and it goes to 100.’ And I'm thinking, ‘Wow, I didn't realize that I've been living in this much pain this long with my hip.’”* (P20, 49 y.o. Female, Hip, Non-Coaching, Surgery)
	Reflecting health status	*“I was kind of surprised on some of them that I was so close to the green. And others I was thinking I was more in the red, but I wasn’t… I don’t know what somebody else feels like. [laughs] How can anybody really know? And so, people have told me I have a great tolerance for pain. Maybe I do, and so, that’s why it’s not showing up.”* (P24, 75 y.o. Male, Knee, Non-Coaching, Alternative)
Fostering Communication Between Patient and Surgeon	Setting expectations	*“I would say that the physician was very realistic, the surgeon, and I appreciated that. [The surgeon] said, ‘This is a big improvement for you most likely, but nothing’s perfect.’ And [the surgeon] tried to temper expectations, which was appropriate after seeing this data.”* (P19, 55 y.o. Male, Knee, Coaching, Surgery)
	Asking and answering questions	*“I was impressed with [the surgeon’s] thoroughness and explanation and his use of this chart to answer a lot of questions I had.… I was impressed how he used the graphics to do it.”* (P15, 74 y.o. Male, Knee, Coaching, Surgery)
	Facilitating shared understanding	*“Because sometimes things are lost between translations and what happens, and just trying to make sure that I'm conveying to the doctors the correct information and they're understanding. And I think [the report] helps with that.… At least say, ‘Yeah, here's what we see with you.’ ‘Here's how I feel.’ So, it's a two-way street.”* (P02, 61 y.o. Female, Hip, Coaching, Alternative)
Building My Confidence and Trust	Gaining confidence regarding treatment outcomes	*“[Page 2] I really, really enjoyed reading. I looked at it carefully. Obviously, it’s not a definite predictor, but it lends to optimism and that helps. I mean this is an emotional time. It’s a scary time.”* (P12, 78 y.o. Female, Knee, Coaching, Surgery)
	Facilitating or affirming treatment decision	*“I was getting irritated about filling out 8,000 forms, *etc*. But I got this [report], and my conclusion was I was doing the right thing [with having surgery]. It was very reassuring. And everybody should have it because even if you’ve already made up your mind, as I had, it still was something that said, ‘Do it. You’re gonna do the right thing, and it’s gonna be successful,’ which is very helpful in terms of how you face it.”* (P16, 82 y.o. Male, Hip, Coaching, Surgery)
	Increasing trust in surgeon	*“Because I just think it’s a great tool for both the patient and the doctor. Rather than pulling out of their back pocket, it’s a lot better to say, ‘Hey, this is a sample of 100,000 people and this is where your age group and your level of injury falls. And so I can say at least statistically that you have a better than 50% chance, or you don’t have as good a chance because of things like your diabetes and stuff.’”* (P15, 74 y.o. Male, Knee, Coaching, Surgery)

Parentheses after quotes reflect each participant’s interview ID, age, gender, involved joint, trial arm, and treatment decision

### Providing information about my health status

The most universal benefit reported by participants was informational. Many valued receiving personalized information about their health status, independent of report use with the surgeon. Some described how the report taught them previously unrecognized facets of current or future health status. For example, one patient shared that the current pain data helped her recognize interconnections between her hip and knee symptoms. Another described how the projected health information helped her understand how her comorbidities would affect her postoperative outcomes.

*“[Future] general health confused me and scared me a little bit. But then I realized [surgery’s] not a cure-all. It's not going to make me 25 again. I have other issues. Like COPD and asthma and stuff and my ankle surgery, which I didn't even think of that played a part in that general physical health.”* (P01, 82 y.o. Female, Hip, Coaching, Surgery)

Other patients described the health status information as confirming what they already knew, with some appreciating the concrete presentation of the data. Additionally, the spectrum of novelty versus confirmation differed between participants for various informational elements (e.g., pain versus function).

*“The first page was pretty helpful just to give something concrete to my pain, to see it measured like that… Every morning, you wake up, and you’re like, ‘Ah shit, it still hurts.’ But this is more concrete.”* (P21, 59 y.o. Female, Hip, Coaching, Surgery)

*“It’s nice to see someone score your pain. But, believe me, you know how much pain you’re in. And so, to me, that’s just a recap of what you already know. The function and physical health part was information that I couldn’t deem myself.”* (P09, 75 y.o. Female, Knee, Non-Coaching, Surgery)

Some participants reported that they valued seeing their PROM scores in the context of a potential range of scores or data from other patients as it gave them a new frame of reference for their health status. Some of these participants shared that the contextualized data validated the reality of their pain. Others reflected on the challenge of recognizing severity of symptoms and dysfunction when living with a chronic condition. They suggested that seeing the contextualized data earlier in the disease process would have highlighted the need to seek care.

*“You don’t wanna be a pain. And I tend to take care of other people. I don’t like accepting help. And I don’t like being the focus. And that’s what it was turning into. And it was just affecting every aspect. So, I think [seeing this information] would’ve brought it home sooner for me.”* (P08, 59 y.o. Female, Hip, Coaching, Surgery)

Despite the perceived informational benefits, a few participants reported co-existing concerns about the nature of the data and its ability to reflect their health. Some patients reported that the scores only reflected a snapshot in time while symptom severity fluctuates or that they may have underrepresented their pain because of high pain tolerance.

*“I think the left hip pain is 44. It should be more like maybe a 36… I think it is a snapshot in time when I took this… It seems like the more active I am during the day, the more pain level I have. So, if I was sitting down after dinner and not moving, my pain would be a little less. So, that’s probably what happened when I took this test.”* (P07, 72 y.o. Male, Hip, Coaching, Surgery)

### Fostering communication between patient and surgeon

About a third of participants discussed the personalized report as supporting communication between patient and surgeon. A few patients described the surgeon using PROM data to engage in conversation about expected postoperative outcomes.

*“[The surgeon] explained that on average most people are around a 50 [on general physical health]. And they don’t expect that number to go up much. It’s not as big of a jump as your hip pain or hip function post-surgery versus pre-surgery.”* (P08, 59 y.o. Female, Hip, Coaching, Surgery)

Some participants also discussed use of the PROM-based report to ask or answer questions during the consultation, with the surgeon using the report to answer patients’ questions or the patient using the report to prepare questions for the surgeon. However, while a few patients described the report information as supporting patient self-advocacy during the clinical consultation, one patient cautioned that it was likely inadequate for increasing confidence to ask questions of the surgeon.

*“[The report] gives [the patient] the information. But I don't know if it says you [as the patient] have every right to ask. It’s really information only.”* (P17, 73 y.o. Female, Knee, Coaching, Surgery)

Some participants also described benefits of the PROM data to facilitate a shared understanding between patient and surgeon. The information could help patients communicate their health status in ways the surgeon could understand. Additionally, they described surgeons’ use of the data to communicate in ways that patients felt their concerns had been heard. One participant even described the potential use of the data to create a shared understanding for future clinical consultations.

*“This way I can tell [the doctors] I’m worse than then, or the pictures will help me, the graphs will help me explain myself for what I’m feeling… When I do go to the doctor’s office, I can use it as a base for my problems. That’s the beginning of it for my left foot.”* (P10, 70 y.o. Female, Knee, Coaching, Alternative)

Discussion of communication-related benefits was often hypothetical versus actual, with patients perceiving advantages for report use even if not used that way in their clinical consultation. Some patients reported that surgeons generally emphasized radiographs and other clinical assessments in determining treatment recommendations, with the report itself not being primary in the conversation.

*“[Reviewing the report] was very brief. Frankly, I think the surgeon was more interested in the X-rays*.” (P22, 78 y.o. Male, Hip, Coaching, Surgery)

However, even when the report was not directly used during the office visit, several patients discussed surgeons’ offering of the report as reflecting a broader patient-centered culture.

### Building my confidence and trust

About half the participants described the PROM data as increasing their confidence and trust related to their treatment outcomes, their treatment decision, and the surgeon. Many of these patients specifically appreciated the future health data, which increased their confidence in their treatment outcomes by decreasing fear and increasing optimistic appraisals. Because predictions were only available for the surgical option, descriptions of confidence in treatment outcomes were limited to surgical participants.

*“It just was so uplifting to me to think that, yeah, I know it’s gonna be really painful for a while, but how [surgery] is gonna impact my life was so wonderful.”* (P25, 70 y.o. Female, Knee, Coaching, Surgery)

Many participants also described using the report to facilitate or affirm the treatment decision based on information about current health status (e.g., how bad it is) and/or future health status (e.g., how good it could be). Several participants acknowledged the surgeon as the expert but appreciated a level of autonomous involvement that the report supported, including coming to a decision after feeling informed and heard. Even participants who had already made a decision reported advantages, with some specifically appreciating the ability to continually refer to the report to affirm their decision. While discussion of increased confidence in the treatment decision was more common amongst those who chose surgery, it was also relevant for those who chose nonoperative options.

*“[Page 2] pretty clearly confirmed my thought that I’m not currently a candidate for the surgery. Because the [current] hip pain and the hip function are still more in the [orange], towards the green areas. So, I think if I were gonna have surgery, I would wait until I get into more of the orange to the reds.”* (P14, 57 y.o. Female, Hip, Coaching, Alternative)

Additionally, some participants described decision making about the surgeon and highlighted the report as increasing their confidence and trust in the surgeon. Specifically, one patient framed the surgeon’s use of report-based data and statistics during the consultation as increasing credibility of treatment recommendations. Others more generally described the report as an indicator of a patient-centered focus that increased their confidence in the surgeon.

*“I think it just gives me more confidence in the doctor because they’re looking to get more information to be able to work with their clients, patients.”* (P02, 61 y.o. Female, Hip, Coaching, Alternative)

## Discussion

Patients described actual and hypothetical benefits of receiving feedback on PROMs in the context of a personalized web-based decision report for THA/TKA, including for those who had already decided to undergo surgery before seeing the surgeon. Specifically, they reported benefits related to information, communication, and confidence, which they positioned within a broader lens of patient-centered care.

Our finding that the report provided or confirmed knowledge aligns with previous patient descriptions of PROM feedback as novel or “repackaging” of familiar information [36]. Specifically, participants’ descriptions highlight osteoarthritis as a chronic condition with gradual decline as acknowledged in the literature [2]. Therefore, as suggested by some participants, patients may benefit from earlier symptom and function monitoring for recognition of treatment needs. Such PROM feedback has potential to impact “readiness” for surgery [37] although the effect on THA/TKA timeliness would need to be examined [38]. Expanded monitoring—particularly beyond clinical encounters—may also address our participants’ concerns related to PROM scores as limited in capturing temporal dimensions of pain and function [39]. Scores that are perceived to accurately reflect health status have been described as foundational for patient acceptability of PROMs for clinical care [40], and repeat PROM measurement may be needed for patients to feel that their health status is adequately represented.

Some participants specifically appreciated contextualization of their scores compared to others, which provided them a new frame of reference for their health status beyond their individual lived experience [41]. Other orthopaedic patients similarly recognized potential benefits of PROMs as a comparison tool [40]. The contextualization in our report was made possible by its web-based design, which enabled integration of the patient’s PROM data with national registry-based data. While our report acknowledged the reference population, it used color coding for comparisons based on patient advisor recommendation, which may have enhanced acceptability. A previous study of a TKA feedback report highlighted patient preferences for implicit (e.g., color coding) versus explicit comparisons to a reference population [8].

Regarding communication, our findings about questions and shared understanding align with previously identified advantages of integrating PROMs into individual patient management to support patients to ask questions or feel cared for [14]. Specifically in this clinical population, feeling informed and listened to are components of positive interactions with health care professionals [1, 42]. As described by our participants, PROM-based reports may help patients articulate and show evidence of their concerns, which can help them feel they are being taken seriously during orthopaedic consultations [43]. However, per one participant’s recognition that information may not translate to self-advocacy, PROM-based feedback reports may need to be coupled with low-resource patient activation interventions (e.g., question-building) to optimize communication benefits [44]. Additionally, participants’ description of surgeons using the PROM data to set expectations relates to the concern of unmet expectations acknowledged within the THA/TKA literature [16, 45, 46]. Patients’ perceived benefits suggest that PROM-based feedback, particularly when combined with predictive analytics, may be a way to improve preoperative counseling through discussion of personalized longer-term health outcomes. The parent trial will evaluate whether this feedback improves patients’ perceptions of the decision-making process, expectation fulfillment, and satisfaction.

Some participants described hypothetical versus actual benefits of PROM feedback for communication because they perceived limited use of the PROM feedback by surgeons compared to other clinical data. While orthopaedic surgeons have expressed concerns about using PROMs for individual patient management [47], participating surgeons were advocates for use per voluntary involvement in the trial. Our findings may reflect challenges with patient recall or misalignment between patients’ and clinicians’ perspectives on how PROM feedback should be used in the clinic, with surgeons using the report in ways unrecognized by patients. There may also be barriers to collaborative point of care use of PROM feedback that need further exploration. Within this trial, surgeons were educated on report interpretation and benefits of shared decisions. However, they were not limited to specific methods of report use within the visit and may benefit from more explicit guidance [20].

Our third finding of building confidence and trust highlights patients’ perceptions of the importance of decreasing fear or anxiety in the decision-making process. In previous research, patients expressed concerns and information needs related to postoperative function and quality of life and trust of physician [48–50]. As identified by our participants, a PROM-based report may help alleviate these concerns. One current challenge is the limited availability of PROM data on non-surgical outcomes, restricting personalized predictions to surgery alone, as in other THA/TKA feedback reports [9, 12]. This may result in decision report imbalance toward surgery, biasing treatment decisions [51]. An interesting finding was patients’ perceived value in using the data to affirm a decision they had already made. Several study sites were academic referral centers, and many participants had already seen local surgeons, received a diagnosis and treatment recommendation, and made a treatment decision before seeing the study surgeon. Additional research is needed to understand patients’ perceived value of using PROM data for decision making throughout the diagnosis and decision-making continuum of hip or knee osteoarthritis.

While our study is strengthened by being multi-site, our findings should be interpreted in the context of the sample’s homogeneity in race, ethnicity, education, and health literacy. Our sample is partially constrained by known disparities related to patients evaluated for THA/TKA [52]. However, racial differences in perceived value of PROM feedback [53] underscore the need for more diverse sampling in future work. Understanding patient perspectives in the context of social determinants of health is imperative to address equity concerns with clinical expansion of PROMs, including differential ability to complete, interpret, and act on PROMs [54, 55]. Disparities must be specifically examined related to digital PROM-based applications as certain subgroups may require additional training and support for their use. Furthermore, our interview sample should not be considered representative of the entire trial population since the level of report exposure we required limited the eligible pool. Our interview volunteers may also have had more positive experiences with the report than non-volunteers, minimizing potential patient concerns or disinterest.

The trial design and interview timing made it challenging to distinguish what benefits the patients perceived from the PROM feedback alone versus discussions with the surgeon or health educator. Since we were unable to complete meaningful comparisons of the report exposure groups (e.g., health educator versus surgeon alone), specific recommendations regarding optimal support for the PROM-based decision report are limited. Instances where the report was received after the office visit may also have biased perspectives of its benefits. Furthermore, while our analysis focuses on patients’ perspectives on receiving PROM-based feedback, the feedback occurred in the context of a specific web-based decision report that included additional data. For example, the predicted 12-month outcomes were designed for the parent trial. While applications incorporating predictions in routine care are growing [11, 56], predicted outcomes are not routinely available in PROM reports in electronic health records. Thus, it is unknown what study findings would translate to other PROM report designs.

## Conclusions

We found that patients with hip or knee osteoarthritis perceived benefits of receiving feedback on PROMs in the context of a personalized web-based decision report. Advantages, such as feeling informed and confident, were seen with or without explicit use of the report with the surgeon, highlighting the potential of patient-facing PROM applications beyond clinical consultations. Research in heterogeneous samples may identify subgroups of patients who would benefit more or less from using these applications. Future research is also needed to refine optimal strategies to present PROM data to patients and support their engagement with the data. However, patients’ overall positive perspectives are promising for continued investigation of digital PROM feedback to support patient-centered care for hip and knee osteoarthritis.

## Supplementary Information

**Additional file 1.** Appendix.

## Data Availability

The datasets used and/or analyzed during the current study are available from the corresponding author on reasonable request. Complete interview transcripts are not publicly available to protect participant confidentiality.

## Abbreviations

PROM: Patient-reported outcome measure
THA: Total hip arthroplasty
TKA: Total knee arthroplasty
