# Supplementary material for: Patients’ perspectives on the benefits of feedback on patient-reported outcome measures in a web-based personalized decision report for hip and knee osteoarthritis
Source: BMC Musculoskelet Disord. 2022 Aug 23;23:806. doi: 10.1186/s12891-022-05764-1 (PMC9395772; doi:10.1186/s12891-022-05764-1)
Supplement: Supplementary file 1 — Additional file 1. Appendix. [file 12891_2022_5764_MOESM1_ESM.pdf]

Franklin PD, Zheng H, Bond C, Lavallee DC. Translating Clinical and Patient-Reported Data to Tailored Shared Decision Reports with Predictive Analytics for Knee and Hip Arthritis. ISOQOL Special Issue: Using feedback tools to enhance the quality and experience of care. 2020.]

A.S.K. Arthritis care through Shared Knowledge

Your Arthritis Profile

(based on your FORCE-TJR survey responses)

ID: 95521
Patient Name:
Latest Survey Date: 11/2/2018

PAIN

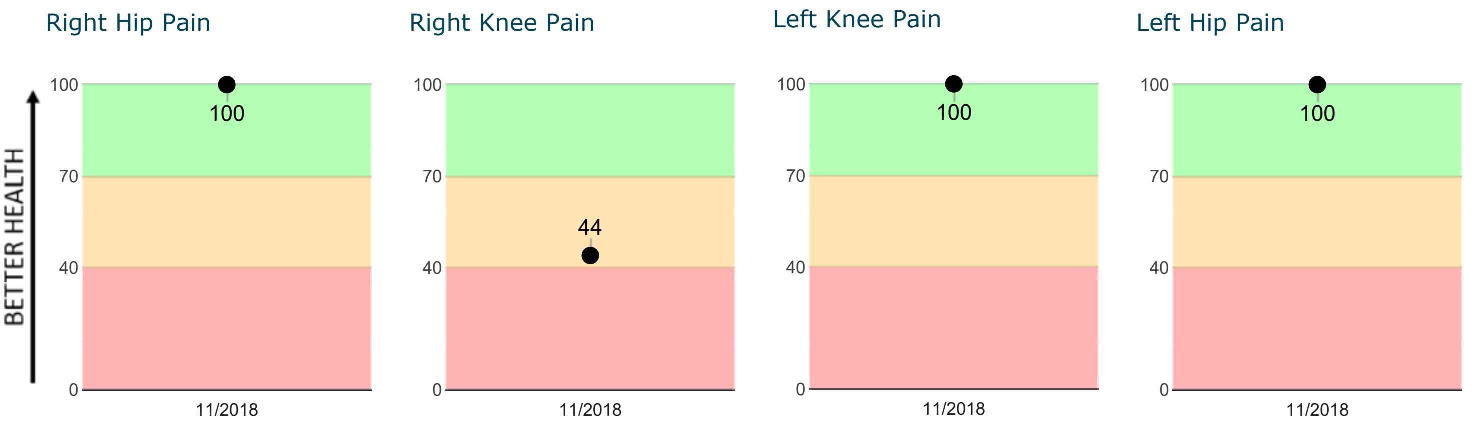

FUNCTION AND PHYSICAL HEALTH

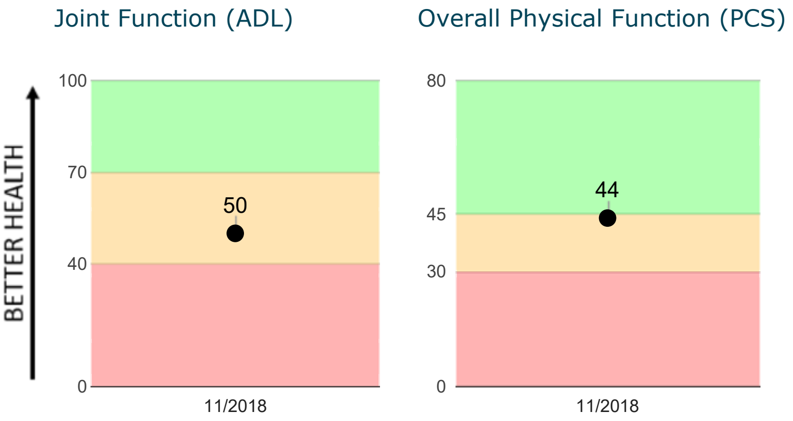

DEFINITIONS AND INTERPRETATION

Pain and Function:
Green: no or mild pain or limitations
Orange: moderate pain or limitations
- discuss treatment options
Red: severe pain or limitations
- surgery is common

Colors and definitions based on the FORCE-TJR nationally representative database

Source:
Pain and ADL values calculated from the HOOS/KOOS
PCS calculated from the VR-12

FACTORS AFFECTING YOUR ARTHRITIS JOINT AND JOINT REPLACEMENT OUTCOMES

Diabetes: Yes

Smoking: No

BMI: 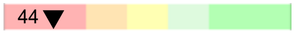

Emotional Health: 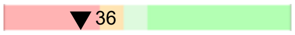

Narcotics Use: No

Low Back Pain: 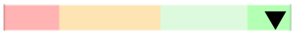

Medical Comorbidity Index: 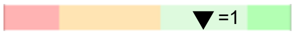

Age: 82

You may be able to improve some of these factors. Please discuss with your surgeon.

Low back Pain:
Green: no pain
Light Green: mild pain
Orange: moderate pain
Red: severe pain

Medical Comorbidity Index:
Green: =0
Light Green: =1
Orange: =2-5
Red: >=6

BMI:
Green: <25
Light Green: 25-30
Yellow: 30-35
Orange: 35-40
Red: >40

Emotional Health:
Green (>50): no limitations
Light Green (45-50): mild limitations
Orange (40-45): moderate limitations
Red (<40): severe limitations

Your Expected Outcomes Based on Patients Like You

(based on FORCE norms)

ID: 95521
Patient Name:
Latest Measure Date: 11/2/2018

Patient's Likely Change in Pain, Function, and Physical Health After Surgery

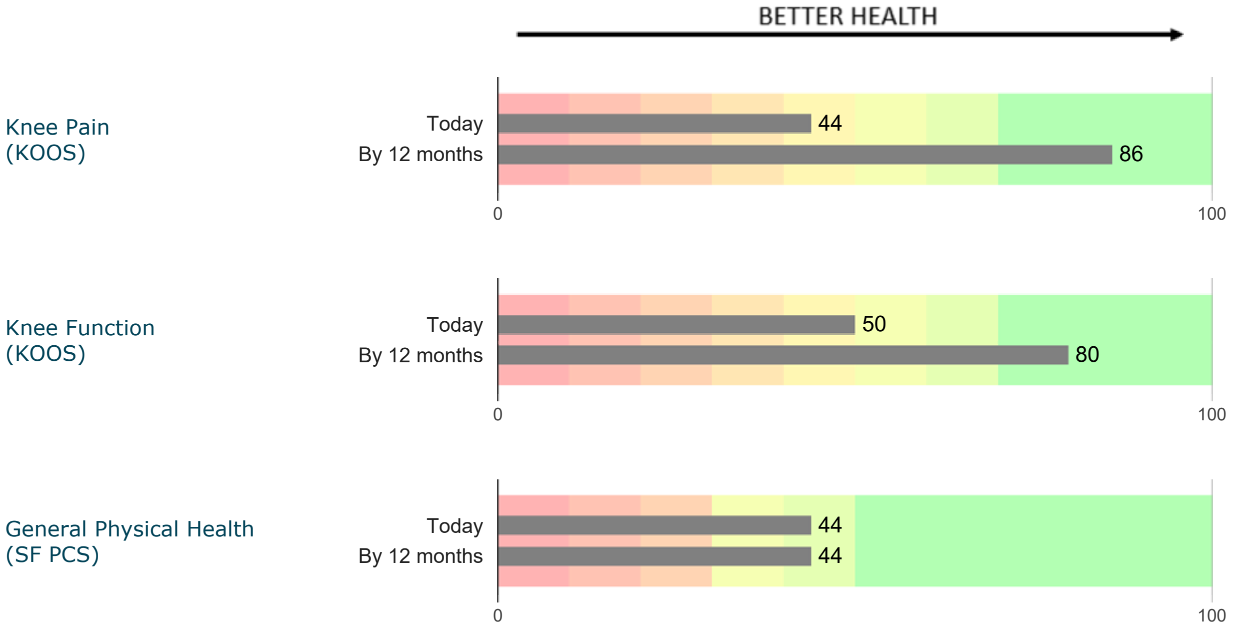

Patient's Likely Pain with Activities of Daily Living After Surgery

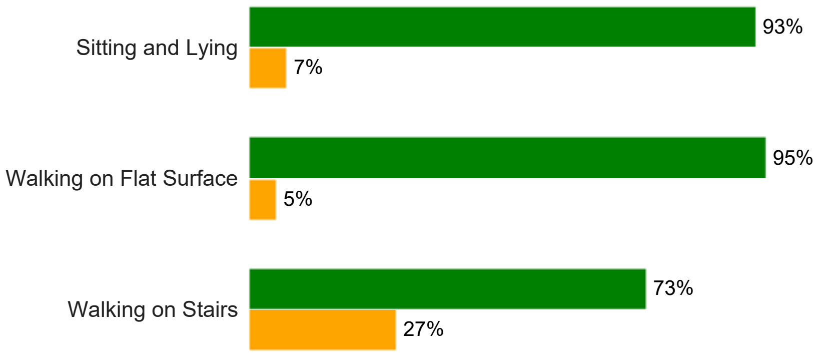

Green: No or Mild Pain

Orange: Moderate or Significant Pain

Patient's Likely Need for Inpatient Care After Surgery

Need for hospital within 30 days National rate: 2% - 8%

Other Treatment Options

ID: 95521
Patient Name:
Latest Measure Date: 11/2/2018

Osteoarthritis of the Knee

Frequently asked questions	Treatment Options Medications for Pain Relief 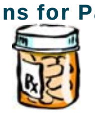	Treatment Options Joint injections (steroids) 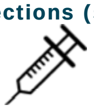	Treatment Options Physical Therapy 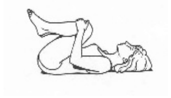
**Will this reduce the pain I have in my knee?**	Ibuprofen (also known as Advil or Motrin) is helpful for 50 in every 100 patients.	Some people get some relief of their pain and/or swelling after an injection.	In a recent study, patients experienced good relief after participating in an intensive physical therapy program that lasted for 12 weeks.
**Will this treatment help improve which activities I can manage to do?**	As you get pain relief, you should be able to be more active.<br><br>Being more active can also help reduce your pain.	If you get pain relief, you may be able to be more active.	As you get pain relief, you should be able to be more active. Most people are able to do more.<br><br>Being more active can also help reduce your pain.
**Are there any risks to this treatment?**	As with all medications, there are some side effects.<br><br>Ibuprofen can cause stomach pain and heartburn.<br><br>It can increase your risk of stomach bleeding.	You might feel slight pain at the injection site for a few days.<br><br>Allergic reactions and infections are rare.<br><br>There is a risk of cartilage damage if you get frequent injections.	You might feel sore for a few days as you begin your physical therapy.<br><br>In a recent study, very few people experienced any medical problems after participating in physical therapy.
**How long will it take me to feel better after the treatment?**	You may start having pain relief within a few days of when you start taking the medication.	Most people who get relief feel better within a week.	It may take up to 4 weeks for you to feel better.
**What are the outcomes for people with arthritis who have this treatment?**	Many people cope well by using medications, being active and losing weight. Reducing your pain may help you get the benefits of exercise.	Some people have good relief when swelling and pain cause problems.	In a recent study, 74 of 100 people who participated in an intensive physical therapy program had not yet had knee replacement after 1 year.
**How long will I need to continue this therapy?**	You will need to stay on this medication until it is no longer working or you decide on another treatment.	You will need to continue this treatment until it is no longer working or you decide on another treatment.	You will need to continue your PT exercises until it is no longer working or you decide on another treatment.
**What is the cost for this treatment?**	These are over the counter medications, so the full cost is yours.	This depends on your insurance and co-pay.	This depends on your insurance and co-pay.
**Important things to know**	You should discuss what pain relief medication might be right for you with your doctor.	In recent studies, it is not clear whether injections are helpful or not. You should discuss whether this would be a good choice for you with your doctor.	You should discuss whether physical therapy would be a good choice for you with your doctor.
**Learn more**	http://www.orthoguidelines.org/topic?id=1005	https://medlineplus.gov/news/fullstory_165630.html	http://www.nejm.org/doi/pdf/10.1056/NEJMoa1505467

Weight Loss can also be helpful in reducing pain and improving knee function. For each pound loss, you decrease the amount of pressure in your knee by 4 pounds. **If you lost 10 pounds, you would decrease the pressure on your knee by 40 pounds.**

Copyright UMass Medical School 2018 . All rights reserved. **Do not copy or distribute without permission.**

Powered by DatStat
